# 

**Published:** 1894-04

**Authors:** 


### Southern Medical Record.

A Monthly Journal of Medicine and Surgery.

VOL. XXIV. ATLANTA, GA., APRIL, 1894. No. 4.

EDITORS:

A. W. GRIGGS, M. D. WILLIS F. WESTMORELAND, M. D.
J. McFADDEN GASTON, M. D. LOUIS H. JONES, M. D.
DAN. H. HOWELL, M. D., BUSINESS MANAGER.

TERMS: $2.00 Per Annum ; Single Copy 20 Cents. Contents on Page 5.

Entered at the Postoffice at Atlanta, Ga., as Second-Class Matter.

The Franklin Printing and Publishing Co., Atlanta, Ga.

